# Evolution, Systematics, and Phylogeography of Pleistocene Horses in the New World: A Molecular Perspective

**DOI:** 10.1371/journal.pbio.0030241

**Published:** 2005-06-28

**Authors:** Jaco Weinstock, Eske Willerslev, Andrei Sher, Wenfei Tong, Simon Y.W Ho, Dan Rubenstein, John Storer, James Burns, Larry Martin, Claudio Bravi, Alfredo Prieto, Duane Froese, Eric Scott, Lai Xulong, Alan Cooper

**Affiliations:** **1** Ancient Biomolecules Centre, Department of Zoology, University of Oxford, United Kingdom,; **2** Institute of Ecology and Evolution, Russian Academy of Sciences, Moscow, Russia,; **3** Department of Ecology and Evolutionary Biology, Princeton University, United States of America,; **4** Government of the Yukon, Cultural Services Branch, Whitehorse, Canada,; **5** Quaternary Paleontology Program, Provincial Museum of Alberta, Edmonton, Canada,; **6** Natural History Museum, University of Kansas, Lawrence, Kansas, United States of America,; **7** Instituto Multidisciplinario de Biologia Celular (IMBICE), La Plata, Argentina,; **8** Instituto de la Patagonia, Universidad de Magallanes, Punta Arenas, Chile,; **9** Department of Earth and Atmospheric Science, University of Alberta, Canada,; **10** San Bernardino County Museum, Redlands, California, United States of America,; **11** China University of Geosciences, Wuhan, China; University of TexasUnited States of America

## Abstract

The rich fossil record of horses has made them a classic example of evolutionary processes. However, while the overall picture of equid evolution is well known, the details are surprisingly poorly understood, especially for the later Pliocene and Pleistocene, c. 3 million to 0.01 million years (Ma) ago, and nowhere more so than in the Americas. There is no consensus on the number of equid species or even the number of lineages that existed in these continents. Likewise, the origin of the endemic South American genus *Hippidion* is unresolved, as is the phylogenetic position of the “stilt-legged” horses of North America. Using ancient DNA sequences, we show that, in contrast to current models based on morphology and a recent genetic study, *Hippidion* was phylogenetically close to the caballine (true) horses, with origins considerably more recent than the currently accepted date of c. 10 Ma. Furthermore, we show that stilt-legged horses, commonly regarded as Old World migrants related to the hemionid asses of Asia, were in fact an endemic North American lineage. Finally, our data suggest that there were fewer horse species in late Pleistocene North America than have been named on morphological grounds. Both caballine and stilt-legged lineages may each have comprised a single, wide-ranging species.

## Abstract 2

Ancient DNA simplifies the systematics of horses present in the New Word during the Pleistocene and reveals that the stilt- legged horse is native to the continent and not a Eurasian migrant.

## Introduction

**Figure 1:**
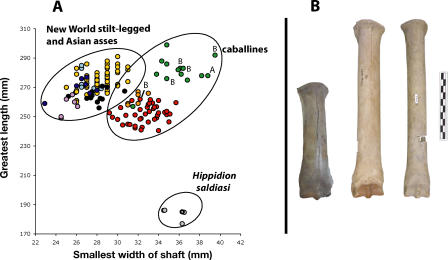
Metatarsal Shape and Size in Pleistocene and Extant Equids (A) Bivariate plot showing metatarsal shape and size in extant and extinct horses. Modern asses: kiang *(E. kiang)* = light blue circles; onager *(E. hemionus onager)* = dark blue; kulan *(E. hemionus kulan)* = purple. Pleistocene equids: stilt-legged from Alaska and the Yukon = black; *E. lambei* (Alaska) = red; stilt-legged from Natural Trap Cave (Wyoming) = yellow; caballines from Natural Trap Cave = orange; caballines from Alberta = green; *H. saldiasi* from southern Patagonia = grey. “A” and “B” above/beside points of caballine horses denote the phylogenetic clade to which the specimens belong (Figure 5). Not all of the specimens were genetically analyzed. (B) Metatarsal shape in Pleistocene horses. From left to right: *H. saldiasi* (southern Patagonia); *E. lambei* (Yukon); NWSL (Yukon). Scale bar is 10 cm.

Nowhere is the later evolution of horses more problematic than in the Americas, where more than 50 species of Pleistocene equids have been named, most of them during the 19th and early 20th centuries. While recent paleontological studies suggest that this number should be drastically revised [1,2], no consensus has been reached about the number of valid species or their phylogenetic relationships. This has limited an in-depth investigation of the biogeography, evolution, and extinction of American Pleistocene equids and their phylogenetic relationships with synchronous Eurasian forms. A particularly problematic example is the so-called North American or New World “stilt-legged” horses (NWSL) found in Mid- and Late Pleistocene deposits in the north and west of North America [1–3]. The NWSL are often found with a second equid form commonly regarded as closely related to the Eurasian caballines, a group that includes the domestic horse *(Equus caballus)* and the extant wild Przewalskii horse. The stilt-legged forms have been taxonomically assigned to a number of species, but all have gracile limbs similar to the extant Asian asses (hemionids—e.g., onager, *E. hemionus;* and kiang, *E. kiang,* Figure 1), leading to suggestions that they are closely related or even result from a dispersal of Eurasian hemionids via the exposed Bering Strait during the last glacial period [4–9]. Alternatively, the NWSL have been regarded as North American endemics [10,11].

**Figure 2:**
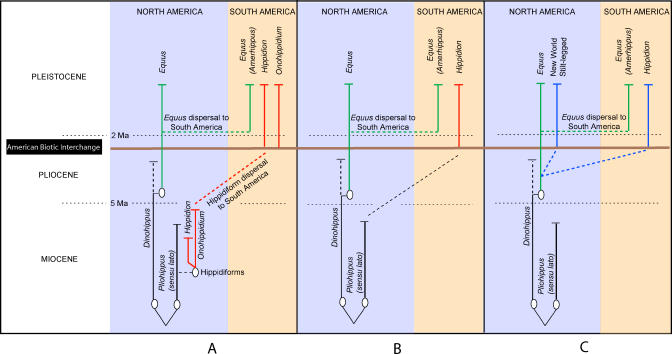
Diagrammatic Representation of North and South American Equid Taxonomy Superimposed on a Time Scale according to Paleontological (A, B) and Molecular Data (C) (A) represents MacFadden's [13] view of hippidiform origins from a pliohippine, diversifying into two genera, *Hippidion* and *Onohippidium,* in North America during the Miocene. (B) shows Alberdi and Prado's [14,15] view of hippidiforms as descendants of a pliohippine during the Miocene; the single genus, *Hippidion*, originates only after dispersal into South America (they do not recognize the genus *Onohippidium* as valid [15]). (C) represents the results of the molecular data presented in the present study; it shows *Hippidion* as originating during the Pliocene, c. 3-3.5 Ma ago. The NWSL is possibly a sister species of *Hippidion*.

Likewise, the systematic position and origins of the South American Pleistocene genus *Hippidion* need to be re-evaluated. This form appeared in South America approximately 2.5 million years (Ma) ago, i.e., shortly after the formation of the Isthmus of Panama enabled the movement of animals between the continents, an event known as the Great American Biotic Interchange [12]. *Hippidion* is considered to be a descendant of the pliohippines, a primitive group of Miocene horses that diverged from the ancestral lineage of equines (a category including all living and extinct members of the genus *Equus,* such as caballine horses, hemiones, asses, and zebras) prior to 10 Ma ago [13−15] (Figure 2). A recent study [16] presented genetic data from three southern Patagonian specimens morphologically identified as *Hippidion*. Unexpectedly, the sequences clustered inside the genus *Equus,* and it was concluded that the bones must be from *E.* *(Amerhippus),* an extinct equine form that dispersed to South America more recently. It was suggested that the bones had been misidentified as *Hippidion* due to (assumed) morphological convergence, but the deep split between *Hippidion* and *Equus* lineages was not questioned. This view is intriguing, as elsewhere in South America where *Hippidion* and *Equus* were sympatric, e.g., central Argentina and southern Bolivia, each form shows well-defined morphological characters in the cranial and postcranial skeleton [17]*.*

## Results/Discussion

**Figure 3:**
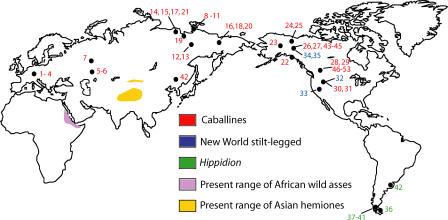
Provenance of Bone Samples Extracted and Sequenced for This Study Details of each sample are given in Table S2.

In order to investigate the phylogenetic position and evolutionary history of the South American hippidiforms and the North American stilt-legged horses, we analyzed a segment between 349 and 716 base pairs (bp) of the mitochondrial control region of fossil equid remains from a number of different localities in North and South America and Eurasia (Figure 3), ranging in date from c. 53,000 years ago to historical times. Mitochondrial DNA was extracted from cortical bone using ancient-DNA techniques and portions of both hypervariable regions (HVR I and II) amplified by PCR in a number of overlapping fragments. Phylogenetic relationships and divergence dates were inferred using maximum likelihood and Bayesian techniques (see Materials and Methods).

**Figure 4:**
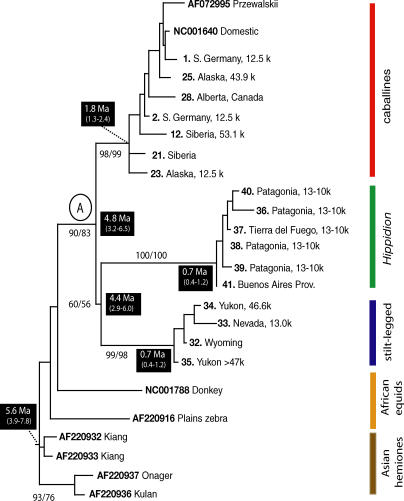
Phylogeny of Recent and Pleistocene Equids The maximum likelihood tree was constructed with two fragments of the mitochondrial control region (583 bp and 133 bp in the HVR1 and HVR2 regions, respectively). Bayesian analysis produced a similar topology. The general time-reversible substitution model was used in both techniques. Black numbers above/beside nodes are posterior probabilities and bootstrap values, respectively (only values > 50% are shown). White numbers on black background are divergence times as calculated from the molecular data. Numbers/letters in bold at the beginning of each specimen's name are sample numbers or GenBank accession numbers. Labels of prehistoric specimens are followed by their age, if available, in thousands of years. The outgroup *(Rhinoceros* and *Ceratotherium)* is not shown.

Phylogenetic analysis using maximum likelihood (Figure 4) resolved *Hippidion,* NWSL, and caballines as three genetically divergent species within a monophyletic group. *Hippidion* and NWSL appear as sister taxa, although the support for this is not high. African zebras and asses and Asian asses (onager and kiang) form a basal polytomy. Bayesian analysis produced a similar topology.

The close phylogenetic relationship between *Hippidion* and caballine horses is in direct contrast to current paleontological models of hippidiform origins. Nevertheless, we are confident that these sequences are those of *Hippidion* rather than the South American caballine form *E. (Amerhippus),* which dispersed into South America about 1 to 1.5 Ma later than *Hippidion*. Morphological studies have concluded that only one species of equid, *H. saldiasi,* is represented in the Late Pleistocene cave deposits of southern Patagonia [18] that generated all but one of our samples. Furthermore, our specimens, mostly phalanges, show not only the typical proportions of *Hippidion*—short and wide—but also morphological, size-independent characteristics typical of this taxon. The first phalanges, for instance, show two short tuberosities for the insertion of the trigonium phalangis; in *Equus,* by contrast, there is a single, long, V-shaped tuberosity [14,17,18]. Finally, both our sequences and those previously reported for three southern Patagonian specimens [16] are virtually identical, and it seems extremely unlikely that all equid DNA sequences from southern Patagonian horses have come from specimens of *E. (Amerhippus)* that have been misidentified as *Hippidion* (contra Orlando et al. [16]). Unfortunately, it was not possible to obtain DNA from any morphologically identified *Amerihippus* specimen to test this, as ancient-DNA survival appears relatively rare in northern South America.

Given the position of *Hippidion* in the tree (Figure 4), its origin postdates the more basal split at node A, estimated at 4.8 Ma (Table S1). Therefore, *Hippidion* should not be seen as a descendant from the Miocene pliohippines; instead, its origins appear to be much more recent, probably during the last stages of the Pliocene (c. 3 Ma), which is close to the time of the first fossil occurrence of this genus [12,14,17,18]. Furthermore, this later date is in opposition to the claim that hippidiforms (genera *Hippidion* and *Onohippidium*) were present in North America as early as the late Miocene (c. 7–8 Ma) [13,19].

Figure 4 also resolves the phylogenetic position of the NWSL as North American endemics rather than Eurasian migrants. Interestingly, specimens from both north (Alaska/Yukon) and south (Wyoming/Nevada) of the Pleistocene ice sheets clearly belong to the same taxon. The apparent large geographic distribution of this species raises the possibility that other Late Pleistocene NWSL currently described as different species—e.g., *E. francisci, E. tau, E. quinni, E. cf. hemionus, E. (Asinus) cf. kiang,* and others with similar limb proportions [1,2,20]—may in fact represent the same taxon. The origins of the NWSL probably lie south of the Pleistocene ice sheets, where Mid-Pleistocene remains (c. 0.5 Ma) with similar limb characteristics have been found [1,2] and Late Pleistocene specimens occur in high frequencies. North of the ice sheets, NWSL remains are relatively rare and appear to have had a restricted temporal distribution [21]. In spite of their presence in eastern Beringia (unglaciated Alaska/Yukon), the stilt-legged horses apparently failed to disperse through the Bering land-bridge into western Beringia (northeast Siberia). In addition, the extinctions of the NWSL north and south of the ice sheets clearly took place at quite different times. Populations in eastern Beringia disappeared around 31,000 years ago [21], while, as demonstrated by the AMS date from the Nevada specimen, NWSL persisted south of the ice sheets until at least 13,000 years ago before present (Table S2), close to the date of the other megafaunal extinctions [4,21].

The phylogenetic patterns and divergence dates in Figure 4 suggest that the morphological apomorphies developed relatively rapidly in both NWSL and *Hippidion*. The greatly retracted nasal notch and long nasal bones that define *Hippidion* [14,15] apparently originated between 4.4 Ma and the late Pliocene–early Pleistocene (c. 2–2.5 Ma), when *Hippidion* first appears in the fossil record [12,15]. The extreme divergence in distal limb proportions between NWSL and *Hippidion* would have occurred during the same interval and presumably reflects adaptations to particular environments [22]. The similar proportions of metapodials in NWSL and Eurasian hemiones are likely to be convergent cursorial adaptations to arid environments with hard soils and scarce vegetation.

**Figure 5:**
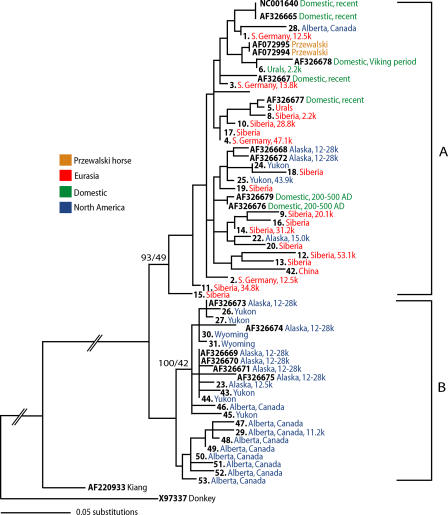
Maximum Likelihood Phylogenetic Tree of Recent, Historic, and Pleistocene Caballine Horses Based on 364 bp of the Mitochondrial Control Region (HVR1) Posterior probabilities/bootstrap values are presented above or beside the main nodes only if either is > 50%. Labels of prehistoric specimens are followed by their age, if available, in thousands of years (k).

A third significant discovery from Figure 4 is that all caballine horses from western Europe to eastern Beringia—including the domestic horse—are a single Holarctic species. A more detailed phylogeographic analysis was performed using only 349 bp of the HVR1 region in order to be able to include a larger number of sequences of Pleistocene, historic, and recent caballines (Figure 5). Little phylogeographic structure is apparent, probably reflecting the high degree of mobility and adaptability of this species. A recent study showed that caballines group into two major clades [23]. Figure 5 confirms this and shows that one clade (A) was broadly distributed from central Europe to North America north and south of the ice. The second clade (B), however, seems to have been restricted to North America. If present in the Old World at all, it probably disappeared there before horse domestication took place, around 5,000 years BP, as all domestic horses—only some of which are shown in the tree—cluster within clade A.

Size has been used as one of the main morphological criteria for defining species of Pleistocene equids [1], and the body size of the Late Pleistocene North American caballines we sampled did exhibit marked regional variability; e.g., horses in Alberta (Canada) were larger than their eastern Beringian and Wyoming counterparts (see Figure 1). The DNA evidence strongly suggests, however, that all of these large and small North American caballine samples belong to a single species (Figure 5 and Table S3). The presence of a morphologically variable caballine species widely distributed north and south of the Pleistocene ice sheets raises the tantalizing possibility that, in spite of the many taxa named on morphological grounds [1,2,4], most or even all North American caballines were members of the same species.

Thus, our results indicate that only two lineages—a caballine and a stilt-legged—may have been present in North America during the Late Pleistocene, each comprising perhaps only a single species with temporal and regional variation in body size and morphology. This model would greatly simplify the systematics of North American Pleistocene horses and could open the way to the analysis of morphological variation in terms of adaptation to different environments. The study of this variation, in combination with paleoclimatic and paleoenvironmental data, should dramatically improve our understanding of the biogeography, evolution, and extinction of horses in this continent.

## Materials and Methods

### DNA extraction, amplification, and sequencing

Details on the provenance and radiocarbon date (where available) of the specimens are given in Table S2. Samples (approximately 0.3–0.5 g) were removed from sections of cortical bone with a Dremel (Racine, Wisconsin, United States) tool, and the outermost layers removed by abrasion. These were then ground to a powder in a Braun (Melsungen, Germany) Mikrodismembrator and decalcified in 10–30 vol of 0.5 M EDTA (pH 8) overnight at room temperature. The sediment was collected by centrifugation and digested in 6 ml of extraction buffer containing 10 mM Tris-HCl (pH 8), 10 mM NaCl, 0.5 mg/ml proteinase K, 10 mg/ml dithiothreitol, 1% sodium dodecyl sulphate, and 0.001–0.01M N-phenacylthiazolium bromide. The extraction was incubated overnight at 45–50 °C. Following digestion, the extraction was added to an equal volume of Tris-saturated phenol and rotated constantly for 10 min before centrifugation at 6,000 rpm for an additional 10 min. The aqueous phase was then removed. This step was repeated twice, once with an equal volume of phenol and then with an equal volume of chloroform. DNA was desalted and concentrated to c. 100 μl using Centricon-30 (Amicon [Millipore, Billerica, Massachusetts, United States]) devices. DNA extractions were performed in batches of six to 14 samples, with one to two negative extraction controls (no bone powder) used for every run.

PCR amplifications were performed in a 25-μl reaction using 1 μl of extract, 1.25 U Platinum *Taq* Hi-Fidelity and 10X buffer (Invitrogen, Carlsbad, California, United States), 2 mg/ml BSA, 2 mM magnesium sulfate, 250 μM of each DNTP, and 1 μM of each primer. DNA was amplified in a MTR Tetrad under the following cycling conditions: 40 cycles of 45-s denaturation at 94 °C, 45-s annealing at 56–63 °C (depending on primer; Table S4), and 90-s extension at 68 °C with a final extension of 10 min at 68 °C. Negative extraction controls and negative PCR controls were used in each PCR reaction. A 583-bp contiguous segment of the HVR1 of the mitochondrial control region and a 133-bp segment of the HVR2 DNA were amplified using overlapping fragments (Table S4).

Sequencing reactions (10 μl) were performed on both strands using PRISM BigDye Terminator v3.1 Cycle Sequencing Ready Reaction Kit (Applied Biosystems, Foster City, California, United States). After the removal of unincorporated dye terminators and primers through ethanol precipitation, PCR products were sequenced on an ABI 310 automated sequencer (PerkinElmer, Wellesley, California, United States) at the DNA-sequencing facility of the Department of Zoology, University of Oxford.

All procedures up until PCR amplification were performed in the specialist Henry Wellcome Ancient Biomolecules Centre, while amplification, characterization, and sequencing took place in the physically distant Department of Zoology to minimize contamination risk [24]. In order to evaluate template damage, cloning of six individual PCR products was performed (12 clones per product) using the Invitrogen Topo-TA cloning kit (see Table S2). Cloned products showed only limited amounts of sequence modification due to template damage [25,26]. In order to verify the authenticity of the sequences, four samples were independently extracted and sequenced at the ancient DNA laboratory at the University of Copenhagen. These included two stilt-legged equids (YG130.3 and YG109.7) and two caballine horses (IEM-202–128 and P94.1.415). Sequences were aligned manually using Se-Al v.2.0a11 [27].

Phylogenetic analyses used maximum likelihood and Bayesian Markov chain Monte Carlo (MCMC) methods in PAUP 4.0 and MrBayes 3.0 software packages, respectively [28, 29]. In the Bayesian analysis, the posterior probability distribution of trees was approximated by drawing a sample every 500 steps over 5,000,000 MCMC cycles, after discarding a burn-in of 500,000 cycles. In both likelihood and Bayesian MCMC analyses, a GTR+G+I model of nucleotide substitution was used. Two species of rhino, *Ceratotherium simum* and *Rhinoceros unicornis,* were used as outgroups.

### Metrical data

Metrical data were obtained from stilt-legged and caballine equid metatarsals from Natural Trap Cave, Wyoming (University of Kansas, United States), from Alaska (Frick Collection, AMNH New York, United States), Alberta (Provincial Museum of Alberta, Canada), and specimen YG150.82 from Thistle Creek (Yukon, Canada). Measurements were taken to the nearest 0.1 mm, following Eisenmann and Bekouche [30]. Additional data for stilt-legged horses from the Yukon and for recent Eurasian asses were obtained from published sources [30,31]. Metrical data of *H. saldiasi* were gathered from published sources [32] and from a specimen from Mylodon Cave, Chile, in the collection of the Museo de la Plata (MLP-6–455).

### Divergence date estimation

The ages of the most recent common ancestors (MRCAs) of *Equus,* stilt-legged horses, and caballine horses, along with the date of the caballine–hippidiform split, were estimated using Bayesian inference as implemented by the program BEAST [33,34]. This program can accommodate non-contemporaneous sequences. The date estimates were made under a general time-reversible model of nucleotide substitution. Rate variation among sites was modelled using a discrete gamma distribution with eight rate categories, and the proportion of invariant sites was estimated.

Monophyly was enforced for the clades only when the ages of MRCAs were being estimated; otherwise, no restrictions were placed on the topology. Consequently, the divergence dates produced by the program have been integrated over the various tree topologies sampled throughout the MCMC analysis, and weighted in proportion to their probability of occurrence. This implies that topological uncertainty has been factored into the divergence date estimates.

The program BEAST incorporates the ages of sequences derived from carbon dating into the estimation of divergence dates. However, because the ages of the sequences are considerably younger than the internal nodes of the tree, it is preferable to provide further calibration information in order to avoid the need for large extrapolations. Accordingly, an additional calibration point was provided by placing a normal prior, with mean 3.0 Ma and standard deviation 0.5 Ma, on the age of the MRCA of *Hippidion* and the stilt-legged horses. This calibration is based on the formation of the Panama isthmus, which has been estimated at 2.5 to 3.0 Ma old [12]. In order to accommodate the possibility that dispersal across continents occurred prior to the complete formation of the isthmus, we repeated the analysis with values of 4.0 Ma and 0.25 Ma for the mean and standard deviation, respectively. The results of these analyses were cross-validated by performing a third analysis, using a normal prior with mean 55 Ma and standard deviation 5 Ma, on the age of the MRCA of rhinoceroses and horses. In all three analyses, a rigid lower bound of 1.3 Ma was placed on the age of the caballine clade, based on fossil evidence [20].

The posterior distributions of the dates being estimated were approximated by sampling parameter values at every 250th cycle over 10,000,000 MCMC steps, after discarding 1,000,000 burn-in steps. The starting tree for the MCMC analysis was generated using a coalescent process. Convergence of the sampled parameters was checked using the program Tracer [35], which revealed that the effective sample sizes of the sampled parameters were well in excess of 100, the minimum recommended effective sample size (AJ Drummond, personal communication).

## Supporting Information

Table S1 Estimated Divergence Times of Various Clades Using Bayesian Inference as Implemented by the Program BEAST (59 KB DOC).

Table S2 Specimens Extracted for This Study (99 KB DOC).

Table S3 Intra- and Inter-Clade HKY+G+I-Corrected Average Pairwise Distance in Equid Clades (Based on 349 bp of the HVR1 Control Region) (21 KB DOC).

Table S4 Primers Used in the Amplification of mtDNA, Control Region (24 KB DOC).

### Accession Numbers

The GenBank (http://www.ncbi.nlm.nih.gov/Genbank) accession numbers for genes and gene products discussed in this paper are: *C. simum* (NC001808), *R. unicornis* (NC001779), and the segment between 349 and 716 bp of the mitochondrial control region of fossil equid remains from a number of different localities in North and South America and Eurasia (DQ007552–DQ007621).

## Abbreviations

bp: base pairs
HVR: hypervariable region
Ma: million years
MCMC: Markov chain Monte Carlo
MRCA: most recent common ancestor
NWSL: New World “stilt-legged” horses.
