# Supplementary material for: Evolution, Systematics, and Phylogeography of Pleistocene Horses in the New World: A Molecular Perspective
Source: PLoS Biol. 2005 Jun 28;3(8):e241. doi: 10.1371/journal.pbio.0030241 (PMC1159165; doi:10.1371/journal.pbio.0030241)
Supplement: Supplementary file 1 [file pbio.0030241.st001.doc]

**Table S1**: Estimated divergence times of various clades using Bayesian inference as implemented by the program *BEAST* (Drummond AJ, Nicholls GK, Rodrigo AG, Solomon W (2002) Genetics 161: 1307; Drummond AJ, Rambaut A (2003) *Beast* v1.2. Available from <http://evolve.zoo.ox.ac.uk/beast/>). We used three different calibration points (A-C). Results from ‘C’ were used in Figure 4 in the main text.

| **Calibration point used** | Event being dated | **Mean** | **95% Credibility interval** | |
| --- | --- | --- | --- | --- |
|  |  |  | lower | **upper** |
|  |  | Million years (Ma) BP | | |
| A |  |  | | |
| Formation of Panamanian isthmus at 3.0 +/- 0.5 | Horse/Rhino split | **41.3** | 25.2 | 59.1 |
|  | Horse clade-root | **4.2** | 3.2 | 5.3 |
|  | *Hippidion*/Stilt-legged clade-root | **3.2** | 2.6 | 3.8 |
|  | *Hippidion* clade-root | **0.58** | 0.34 | 0.82 |
|  | Caballines clade-root | **1.4** | 1.3 | 1.7 |
|  | Caballines/*Hippidion*+stilt-legged clade-root | **3.5** | 2.8 | 4.3 |
|  | Stilt-legged clade-root | **0.60** | 0.32 | 0.90 |
| B |  |  |  |  |
| Formation of Panamanian isthmus at 4.0 +/- 0.25 | Horse/Rhino split | **50.2** | 31.0 | 70.9 |
|  | Horse clade-root | **5.1** | 4.1 | 6.3 |
|  | *Hippidion*/Stilt-legged clade-root | **4.0** | 3.6 | 4.3 |
|  | *Hippidion* clade-root | **0.71** | 0.42 | 1.0 |
|  | Caballines clade-root | **1.6** | 1.3 | 2.1 |
|  | Caballines/*Hippidion*+stilt-legged clade-root | **4.4** | 3.7 | 5.1 |
|  | Stilt-legged clade-root | **0.71** | 0.39 | 1.0 |
| C |  |  |  |  |
| Rhino-horse divergence at 55 +/- 5 | Horse/Rhino split | **54.9** | 51.4 | 58.3 |
|  | Horse clade-root | **5.6** | 3.9 | 7.8 |
|  | *Hippidion*/Stilt-legged clade-root | **4.4** | 2.9 | 6.0 |
|  | *Hippidion* clade-root | **0.76** | 0.45 | 1.2 |
|  | Caballines clade-root | **1.8** | 1.3 | 2.4 |
|  | Caballines/*Hippidion*+stilt-legged clade-root | **4.8** | 3.2 | 6.5 |
|  | Stilt-legged clade-root | **0.77** | 0.40 | 1.2 |
