# Supplementary material for: Evolution, Systematics, and Phylogeography of Pleistocene Horses in the New World: A Molecular Perspective
Source: PLoS Biol. 2005 Jun 28;3(8):e241. doi: 10.1371/journal.pbio.0030241 (PMC1159165; doi:10.1371/journal.pbio.0030241)
Supplement: Supplementary file 2 [file pbio.0030241.st002.doc]

## Table S2. Bone specimens extracted for this study. Sequences were submitted to GenBank (accession numbers DQ007552-DQ007621).

| **Sample no.** | **Specimen no.** | **Extract**  **code** | **Morph. species identification** | **Element** | **Provenance** | **AMS date**  **(BP)** | **Sequence length (bp)** |
| --- | --- | --- | --- | --- | --- | --- | --- |
| 1 | PET09^1^ | JW177 | Equus sp. | Astragalus | Petersfels, Germany | 12,545±50 | 716 |
| 2 | SMNS^2^ | JW175 | Equus sp. | Astragalus | Hohlefels, Germany | 12550±60 | 716 |
| 3 | Vog IV-6671^3^ | JW266 | *Equus* sp. | Radius/Ulna | Vogelherd IV, Germany | 13.845±50 | 349 |
| 4 | SMNS 8010^2^ | JW196 | *Equus* sp. | Metatarsal | Bockstein, Germany | 47,100±1000 | 349 |
| 5 | Ek 253/557^4^ | JW202 | *Equus* sp. | Calcaneus | Ignatievskaya, Ural Mts. |  | 349 |
| 6 | EK 253/556^4^ | JW16 | *Equus* sp. | Phalange II | Ignatievskaya, Ural Mts. | 2,218±34 | 349 |
| 7 | EK 994/217^4^ | JW17 | Equus sp. | Phalange I | Sur’ya 5, Ural Mts. |  | 349 |
| 8 | IEM 202-279^5^ | JW25 | *Equus* sp. | Pelvis | Bol. Lyakhovsky Island, N-E Siberia | 2,220±50* | 349 |
| 9 | IEM 202-128^5^ | JW27 | *Equus* sp. | Humerus | Bol. Lyakhovsky Island, N-E Siberia | 20,100±170* | 349 |
| 10 | IEM 202-847^5^  ^Indep. replicated^ | JW28 | *Equus* sp. | Femur | Bol. Lyakhovsky Island, N-E Siberia | 28,800±1100* | 349 |
| 11 | IEM202-244^5^ | JW29 | *Equus* sp. | Pelvis | Bol. Lyakhovsky Island, N-E Siberia | 34800±1000* | 349 |
| 12 | PIN 3659-6^6^ | JW188 | Equus sp. | Metatarsal | Ulakhan-Sullar, Adycha R., Yana Basin, N-E Siberia | 53,100±1700 | 716 |
| 13 | PIN 3659-1^6^ | JW189 | *Equus* sp. | Metatarsal | Ulakhan-Sullar, Adycha R., Yana Basin, N-E Siberia |  | 349 |
| 14 | IEM203-42^5^ | JW190 | *Equus* sp. | Humerus | Lena R. Delta, N-E Siberia | 31220±180 | 349 |
| 15 | IEM 205-6^5^ | JW191 | *Equus* sp. | Tibia | Bykovsky Peninsula, Lena Delta, NE Siberia |  | 349 |
| 16 | PIN 3658-121^6^ | JW194 | *Equus* sp. | Humerus | Alyoshkina, Kolyma R., N-E Siberia |  | 349 |
| 17 | IEM 207-30^5^ | JW195 | *Equus* sp. | Ulna | Lena R. Delta, N-E Siberia |  | 349 |
| 18 | PIN 169-43^6^ | JW209 | *Equus* sp. | Metacarpal | Kolyma R. lower course, N-E Siberia |  | 349 |
| 19 | PIN 3751-151^6^ | JW207 | *Equus* sp. | Metatarsal | Yana R. Lower course, N-E Siberia |  | 349 |
| 20 | PIN 3100-421^6^ | JW210 | *Equus* sp. | Metatarsal | Chukochya R., Kolyma Lowland, N-E Siberia |  | 349 |
| 21 | IEM 200-483^5^ | JW203 | Equus sp. | Scapula | Bykovsky Peninsula, Lena Delta, N-E Siberia | 27,500±400* | 716 |
| 22 | UA-97-061-221^7^ | JW69 | *Equus* sp. | Radius | Castle River, Alaska | 15,090 | 349 |
| 23 | AA26819^8^ | JW184 | Equus sp. | Metacarpal | Ester Creek, Alaska | 12510±130 | 716 |
| 24 | CMN MgVo-3-85-60^9^ | JW78 | Equus sp. | Metatarsal | Bluefish Cave, Yukon |  | 349 |
| 25 | CMN 49368^9^ | JW98 | Equus sp. | Metacarpal | Old Crow Loc. 22, Yukon | 43,900±180 | 716 |
| 26 | YG110.111^10^ | JW128 | Equus sp. | Metacarpal | Irish Gulch, Yukon |  | 716 |
| 27 | YG150.82^10^ | JW129 | Equus sp. | Metatarsal | Thistle Creek, Yukon |  | 349 |
| 28 | P94.1.415^11^  ^Indep. replicated^ | JW71 | Equus sp. | Metatarsal | Edmonton Pit 48, Alberta |  | 716 |
| 29 | P89.21.1^11^ | JW174 | Equus sp. | Femur | Grand Prairie, Alberta | 11,200±90 | 349 |
| 30 | KU42625^12^ | JW157 | Equus sp. | Metatarsal | Natural Trap Cave, Wyoming |  | 349 |
| 31 | KU51467^12^ | JW160 | Equus sp. | Humerus | Natural Trap Cave, Wyoming |  | 349 |
| 32 | KU62158^12^ | JW161 | *Equus* sp. | Metatarsal | Natural Trap Cave, Wyoming |  | 716 |
| 33 | LACM 109/ 150807^13^  ^Cloned^ | JW277 | *Equus* sp. | Femur | Gypsum Cave, Nevada | 13070±55 | 716 |
| 34 | YG130.3^10^  ^Indep. replicated^ | JW125 | *Equus* cf. *kiang* | Phalange I | Quartz Creek, Yukon | 46600±1000 | 716 |
| 35 | YG109.7^10,^  ^Indep. replicated,^  ^Cloned^ | JW126 | *Equus* cf. *kiang* | Metatarsal | Quartz Creek, Yukon | > 47000 | 716 |
| 36 | TA 14001^14^ | JW257 | Hippidion saldiasi | Phalange I | Tierra del Fuego, Chile |  | 599 |
| 37 | CLS1 44887^14^  ^Cloned^ | JW245 | Hippidion saldiasi | Phalange II | Cueva Lago Sofia 1, south. Patagonia, Chile |  | 716 |
| 38 | CLV, n.a. ^14^  ^Cloned^ | JW256 | Hippidion saldiasi | Phalange II | Cueva de la Ventana, south. Patagonia, Chile |  | 716 |
| 39 | CLS1 42563^14^ | JW260 | Hippidion saldiasi | Phalange I | Cueva Lago Sofia 1, south. Patagonia, Chile |  | 716 |
| 40 | CM 28/6c^14^ | JW244 | Hippidion saldiasi | Phalange II | Cueva del Medio, south. Patagonia, Chile |  | 716 |
| 41 | MLP 6-272^15^ | JW251 | Hippidion principale | Metatarsal | Provincia de Buenos Aires, Argentina |  | 716 |
| 42 | HS23^16^ | JW163 | Equus sp. | Metatarsal | Tonghe Bridge, Harbin, China |  | 349 |
| 43 | YT03/280^10^  ^Cloned^ | JW119 | Equus sp. | Radius | Thistle Creek, Yukon |  | 349 |
| 44 | YT03/286^10^  ^Cloned^ | JW123 | Equus sp. | Radius | Thistle Creek, Yukon |  | 349 |
| 45 | YT03/279^10^ | JW120 | Equus sp. | Skull | Thistle Creek, Yukon |  | 349 |
| 46 | P97.12.9^11^ | JW172 | Equus sp. | Metacarpal | Cloverbar sand and gravel, Edmonton, AB |  | 349 |
| 47 | P98.6.8^11^ | JW170 | Equus sp. | Metatarsal | Cloverbar sand and gravel, Edmonton, AB |  | 349 |
| 48 | P96.2.33^11^ | JW218 | Equus sp. | Metacarpal | Inland Pit 48, Edmonton, AB |  | 349 |
| 49 | P96.9.18^11^ | JW222 | Equus sp. | Scapula | Twin Bridges Gravel Pit 80, Edmonton, AB |  | 349 |
| 50 | P88.19.3^11^ | JW219 | Equus sp. | Metatarsal | Apex Evergreen Pit, Edmonton, AB |  | 349 |
| 51 | P95.1.12^11^ | JW220 | Equus sp. | Metatarsal | Cloverbar sand and gravel, Edmonton, AB |  | 349 |
| 52 | P94.2.1^11^ | JW221 | Equus sp. | Metacarpal | Twin Bridges Gravel Pit 80, Edmonton, AB |  | 349 |
| 53 | P95.2.29^11^ | JW216 | Equus sp. | Metatarsal | Apex Evergreen Pit, Edmonton, AB |  | 349 |
| PF | KU819377^10^ | JW74 | Equus sp., stilt legged | NA | Natural Trap Cave, Wyoming | 27410±200 | 165 |
| PF | AA26786^8^ | JW181 | *Equus* sp., stilt-legged | Metacarpal | Cripple Creek, Alaska | 33600±1700 | 349 |
| PF | KU62055^12^ | JW158 | Equus sp. | Metatarsal | Natural Trap Cave, Wyoming |  | 249 |
| PF | YG109.7^10^ | JW126 | *Equus* sp., stilt-legged | Metatarsal | Quartz Creek, Yukon | > 47000 | 349 |
| F | AA26787^8^ | JW182 | *Equus* sp., stilt-legged | Metacarpal | Ester Creek, Alaska | 32600±1400 | -- |
| F | AA26796^8^ | JW183 | *Equus* sp., stilt-legged | Metacarpal | Cripple Creek, Alaska | 40800±3900 | -- |
| F | MLP-6-456^15^ | JW246 | *Hippidion saldiasi* | Metacarpal | Mylodon Cave, Chile |  | -- |
| F | MLP-6-455^15^ | JW247 | *Hippidion saldiasi* | Metatarsal | Mylodon Cave, Chile |  | -- |
| F | MLP-6-643^15^ | JW248 | *Hippidion saldiasi* | Metatarsal | Mylodon Cave, Chile |  | -- |
| F | MLP-81-III-28-7^15^ | JW249 | *Hippidion saldiasi* | Phalange I | Mylodon Cave, Chile |  | -- |
| F | MLP-94-VIII-10-50^15^ | JW250 | *Hippidion saldiasi* | Mandible | Mylodon Cave, Chile |  | -- |
| F | MLP-80-VIII-13-63^15^ | JW252 | *Equus* (*Amerhippus*) | Phalange I | Paso Otero, Prov. Buenos Aires, Argentina |  | -- |
| F | MLP-86-III-25-17^15^ | JW253 | *Equus* (*Amerhippus*) | Phalange I | Arroyo Tapalque, Prov. Buenos Aires, Argentina |  | -- |
| F | SMNS-Ses 04^2^ | JW45 | *Equus* sp*.* | Tibia | Sessefels Cave, Germany |  | -- |
| F | CMN-464978^9^ | JW84 | *Equus verae* | Metatarsal | Sixtymile Loc.3, Yukon |  | -- |
| F | CMN-46502^9^ | JW85 | *Equus verae* | Metatarsal | Sixtymile Loc. 3, Yukon |  | -- |
| F | KU53352^12^ | JW113 | *Equus* sp. | Metatarsal | Natural Trap Cave, Wyoming |  | -- |
| F | KU26470^12^ | JW114 | *Equus* sp. | Metatarsal | Natural Trap Cave, Wyoming |  | -- |

* Conventional dates: A.V. Sher, S.A. Kuzmina, T.V. Kuznetsova, L.D. Sulerzhitsky, *Quat. Sci. Reviews* in press (2004); 1. Landesdenkmalamt Baden-Württemberg, Konstanz, Germany; 2. State Museum of Natural History, Stuttgart, Germany; 3. Institute of Pre- and Protohistory, University of Tübingen, Germany; 4. Institute for Plant and Animal Ecology, Ekaterinburg, Russia; 5. Institute of Ecology and Evolution Russian Academy of Sciences, Moscow, Russia; 6. Paleontological Institute, Russian Academy of Sciences, Moscow, Russia; 7. University of Alaska, Fairbanks, USA; 8. Department of Paleontology, American Museum of Natural History, New York, USA; 9. Canadian Museum of Nature, Ottawa, Canada; 10. Yukon Department of Tourism and Culture, Whitehorse, Canada; 11. Provincial Museum of Alberta, Edmonton, Canada; 12. Natural History Museum, University of Kansas, Lawrence, USA; 13. Los Angeles County Museum, USA; 14. Instituto de la Patagonia, Universidad de Magallanes, Punta Arenas, Chile; 15. Dept. of Vertebrate Paleontology, Museo de La Plata, La Plata, Argentina; 16 X. Gao, Harbin (China) private collection.
