# Supplementary material for: Evolution, Systematics, and Phylogeography of Pleistocene Horses in the New World: A Molecular Perspective
Source: PLoS Biol. 2005 Jun 28;3(8):e241. doi: 10.1371/journal.pbio.0030241 (PMC1159165; doi:10.1371/journal.pbio.0030241)
Supplement: Supplementary file 3 [file pbio.0030241.st003.doc]

**Table S3**. Intra- and Inter-clade HKY+G+I-corrected average pairwise distance in equid clades (based on 349bp of the HVR1, control region). Pairwise distances of the donkey were calculated using 14 sequences (GenBank accession numbers AY69532-AY69538 and AY69543-AY69547) from wild and domestic specimens, which include individuals of the two wild subspecies, *E. asinus asinus* and *E. asinus somalicus*.

While genetic distances can vary between sibling and congeneric species (see e.g., Johns and Avise (1998) MBE 15: 1481-1490), the genetic distances suggest that caballines, *Hippidion*, and NWSL clades are each monospecific.

| Intra-clade* | distance | Inter-clade | distance |
| --- | --- | --- | --- |
| caballines clade A | 0.03241 | caballines (A+B) vs *E. asinus* | 0.18555 |
| caballines clade B | 0.02206 | caballines vs *Hippidion* | 0.15009 |
| caballines A+B | 0.04175 | caballines vs NWSL | 0.15029 |
| donkey (*Equus asinus*) | 0.05499 | caballines A vs caballines B | 0.05496 |

* ‘A’ and ‘B’ refer to clades in Figure 5 in the main text
