# Supplementary material for: Evolution, Systematics, and Phylogeography of Pleistocene Horses in the New World: A Molecular Perspective
Source: PLoS Biol. 2005 Jun 28;3(8):e241. doi: 10.1371/journal.pbio.0030241 (PMC1159165; doi:10.1371/journal.pbio.0030241)
Supplement: Supplementary file 4 [file pbio.0030241.st004.doc]

**Table S4**: Primers used in the amplification of mtDNA, Control Region. Symbols “»” and “«” denote forward and reverse primer, respectively. All primers designed by authors, except L1, H1, L2, H2, L3, H3, (marked with an ‘*’’) published by Vilá, C. et al. (Widespread origins of domestic horse lineages. *Science* **291**, 474-477 (2001)).

| Primer pairs | **Sequence (5’-3’)** | **Region amplified** | **Annealing temp. (°C)** |
| --- | --- | --- | --- |
| L1 »  H1 « | GCCATCAACTCCCAAAGCT*  ACATGCTTATTATTCATGG* | HVR1 | 56 |
| L2 »  H2 « | CCCACCTGACATGCAATAT*  TGTTGACTGGAAATGATTTG* | HVR1 | 56 |
| L3 »  H3 « | TCGTGCATACCCCATCCAA*  CCTGAAGTAGGAACCAGATG* | HVR1 | 56 |
| L4 »  H3 « | CCATGAATAATAAGCATGT  CCTGAAGTAGGAACCAGATG* | HVR1 | 56 |
| EQ168-187F »  EQCR2bii « | CGTGCATTAAATTGTTTGCC  CATGGGAGGTGATATGCGTG | HVR1 | 56 |
| EQCR3ai »  EQCR3bi « | CGTGCATACCCCATCCAAGTC  GAACCAGATGCCAGGTATAG | HVR1 | 56 |
| EQCR1F »  EQCR136R « | TCCTCGCTCCGGGCCCAT  TGTGAGCATGGGCTGATTAGTC | HVR1 | 60 |
| EQCR51F »  EQCR210R « | CTGGCATCTGGTTCTTTCTTCAG  CTTTGACGGCCATAGCTGAGT | HVR1 | 60 |
| EQCR163F »  EQCR296R « | ACTGTGGTTTCATGCATTTG  TTGCTGATGCGGAGTAATAA | HVR1 | 56 |
| EQCRend184F »  EQCRend342R « | ATCTTGCCAAACCCCAAAAACAAG  TCTAGGGGGATGCCTGTCTATGG | HVR2 | 63 |
